# Preconception care: nutritional risks and interventions

**DOI:** 10.1186/1742-4755-11-S3-S3

**Published:** 2014-09-26

**Authors:** Sohni V Dean, Zohra S Lassi, Ayesha M Imam, Zulfiqar A Bhutta

**Affiliations:** 1 Division of Women and Child Health, Aga Khan University Karachi, Pakistan

**Keywords:** preconception, overweight, folic acid, nutrition

## Abstract

**Introduction:**

There is increasingly a double burden of under-nutrition and obesity in women of reproductive age. Preconception underweight or overweight, short stature and micronutrient deficiencies all contribute to excess maternal and fetal complications during pregnancy.

**Methods:**

A systematic review and meta-analysis of the evidence was conducted to ascertain the possible impact of preconception care for adolescents, women and couples of reproductive age on maternal, newborn and child health (MNCH) outcomes. A comprehensive strategy was used to search electronic reference libraries, and both observational and clinical controlled trials were included. Cross-referencing and a separate search strategy for each preconception risk and intervention ensured wider study capture.

**Results:**

Maternal pre-pregnancy weight is a significant factor in the preconception period with underweight contributing to a 32% higher risk of preterm birth, and obesity more than doubling the risk for preeclampsia, gestational diabetes. Overweight women are more likely to undergo a Cesarean delivery, and their newborns have higher chances of being born with a neural tube or congenital heart defect. Among nutrition-specific interventions, preconception folic acid supplementation has the strongest evidence of effect, preventing 69% of recurrent neural tube defects. Multiple micronutrient supplementation shows promise to reduce the rates of congenital anomalies and risk of preeclampsia. Although over 40% of women worldwide are anemic in the preconception period, only one study has shown a risk for low birth weight.

**Conclusion:**

All women, but especially those who become pregnant in adolescence or have closely-spaced pregnancies (inter-pregnancy interval less than six months), require nutritional assessment and appropriate intervention in the preconception period with an emphasis on optimizing maternal body mass index and micronutrient reserves. Increasing coverage of nutrition-specific and nutrition-sensitive strategies (such as food fortification; integration of nutrition initiatives with other maternal and child health interventions; and community based platforms) is necessary among adolescent girls and women of reproductive age. The effectiveness of interventions will need to be simultaneously monitored, and form the basis for the development of improved delivery strategies and new nutritional interventions.

## Introduction

Nutritional status is an important aspect of health and wellness before and during pregnancy. Under nutrition in women contributes to 20% of maternal deaths, and is a significant risk factor for stillbirths, preterm births, small for gestational age and low birth weight babies [1-7], yet in most countries 10-20% of women are underweight [8]. Maternal short stature heightens the risk for obstructed labor, obstetric fistula and maternal mortality, as well as birth asphyxia leading to neonatal death, and is often the result of girls being stunted since childhood [9]. Pre-pregnancy overweight and obesity has been linked to two of the foremost causes of maternal mortality [10,11] - hypertensive disorders of pregnancy [12-15] and gestational diabetes mellitus (GDM) [16,17]- as well as an entire spectrum of adverse pregnancy outcomes [1-7], including poor lactation practices [18,19], obstetric anesthesia-related complications [20], prolonged gestation [21,22], maternal infectious morbidity [23] and decreased success with trial of labor [24-27]. Maternal obesity is a cause for stillbirths, fetal and neonatal death [3,28-31], and moreover, perpetuates the obesity epidemic since children of obese women are more likely to be obese themselves [17,32-36].

In addition to weight, micronutrient status is also linked to pregnancy outcomes. The recent Cochrane review [37] found a strong protective effect (RR 0.28, 95% CI 0.15-0.52) of folic acid on recurrent neural tube defects (NTDs). Other meta-analyses of randomized and observational studies showed a reduction in recurrence risk of 69 to 100% [38] and a reduction in occurrence risk of 42 [39] to 62% [40], yet less than half of all women regularly consume folic acid before conception [41]. Despite research evidence linking iron deficiency with maternal mortality, around 40% of women are anemic globally [9]. Other micronutrients such as zinc and calcium have been found to improve maternal and newborn outcomes when supplementation is provided during pregnancy- it seems likely that ensuring adequate intake of these micronutrients earlier, which is during the preconception period, would be of added benefit for undernourished girls and women and in the case of unplanned pregnancies. Folic acid, B vitamins and zinc have been shown to affect early fetal development, even before women realize they are pregnant. Micronutrient supplementation or fortification is currently being used as strategies to improve nutrition even in resource-poor settings since many girls and women are chronically undernourished [42].

There is a dearth of intervention trials to address under-nutrition or obesity in women of reproductive age. Weight and micronutrient status during pregnancy is influenced by a number of factors such as food insecurity and birth spacing that require broad interventions, hence the aim should to achieve and sustain optimal nutritional intake and weight before pregnancy. In addition, even for women who are overweight or obese, losing weight is not recommended during gestation and therefore weight and nutritional status should be reviewed between pregnancies. Nutritional risks and interventions are an important component of preconception care, defined for the purpose of this review as “any intervention provided to women and couples of childbearing age, regardless of pregnancy status or desire, *before* pregnancy, to improve health outcomes for women, newborns and children” (Detailed discussion of the importance and scope of preconception care is given elsewhere) [43].

This paper presents the findings of a systematic review that was undertaken to consolidate the evidence for nutritional risks before pregnancy, and ascertain the effectiveness of providing interventions during the preconception period (versus periconception or prenatal) on maternal, newborn and child health (MNCH) outcomes. The first section discusses pre-pregnancy weight, which is followed by diet and exercise as interventions to achieve and maintain optimal weight. This is followed by the sections on folic acid, multivitamin and iron supplementation in the preconception period. The review also looks beyond efficacy of an intervention to studies that examined impact of strategies used to increase uptake.

## Methods

We systematically reviewed all literature published up to 2011 to identify studies describing the effectiveness of preconception (period before pregnancy and between pregnancies) nutritional risks and interventions and their impact on maternal, newborn and child health (MNCH) outcomes. Electronic databases such as PubMed, Cochrane Libraries, Embase, and WHO Regional Databases were searched to identify the studies. We included systematic reviews, experimental and observational studies. Papers were also identified by hand searching references from included studies. No language or date restrictions were applied in the search. The findings were presented at international meeting [44,45] and shared with professionals in the relevant fields of maternal and child health, following which results were updated based on current searches (through end of 2012) and expert opinion. Studies were included if they reported the nutritional risks and interventions for MNCH outcomes. Methodology is described in detail elsewhere [43].

For the studies that met the final inclusion criteria, two review authors abstracted data describing study identifiers and context, study design, intervention specifics and outcome effects into a standardized abstraction sheets. The quality of experimental studies were assessed using Cochrane criteria [46], whereas STROBE guidelines were used to assess the quality of observational studies [47]. We conducted meta-analyses for individual studies and pooled statistics was reported as the odds ratio (OR) and relative risk (RR) between the experimental and control groups with 95% confidence intervals (CI). Mantel–Haenszel pooled RR and corresponding 95% CI were reported or the Der Simonian–Laird pooled RR and corresponding 95% CI where there was an unexplained heterogeneity. All analyses were conducted using the software Review Manager 5.1 [48]. Heterogeneity was quantified by Chi^2^ and I^2^, in situations of high heterogeneity, causes were explored and random effect models were used.

## Results

**Figure 1:**
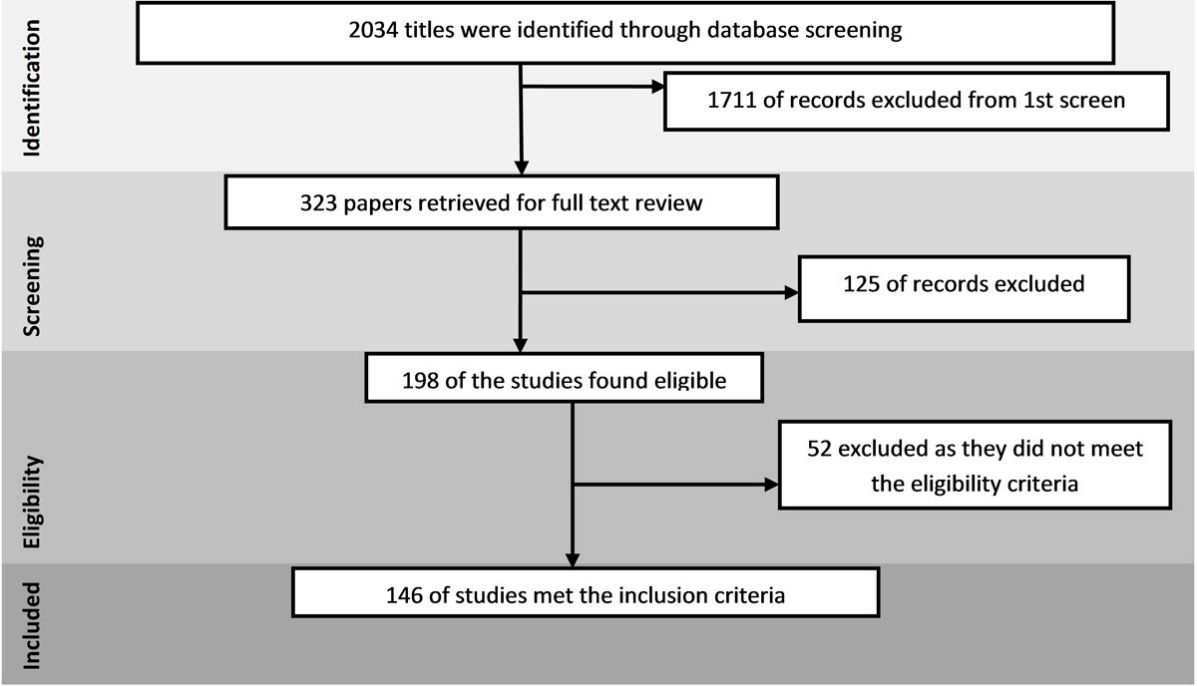
Search flow diagram

The review identified 2034 papers from search in all databases. After the initial title and abstract screening, 198 full texts were reviewed to identify papers which met the inclusion criteria and had the outcomes of our interest. One hundred and forty six studies were finally selected for abstraction and analysis (Figure 1). Information related to each included study can be found on the following link: https://globalmotherchildresearch.tghn.org/site_media/media/articles/Preconception_Report.pdf

## Nutritional risks

### Maternal pre-pregnancy weight

In order to define the categories of weight that are not normal, the World Health Organization and the National Institutes of Health grouped weight into four categories according to individuals’ body mass index: underweight (<18.5 kg/m^2^), normal (18.5–24.9 kg/m^2^), overweight (25.0-29.9 kg/m^2^), and obese (30.0 kg/m^2^) [49]. The literature shows a BMI-dependent relationship between pre-pregnancy obesity and adverse pregnancy outcomes [50,51]. Further, excessive postpartum weight retention is a risk not only for subsequent pregnancies [52,53], but also for the development of maternal chronic diseases. Although guidelines exist for gestational weight gain according to maternal pre-pregnancy BMI, however gestational weight gain is not discussed further as it falls outside the scope of preconception care. Previous reviews have assessed maternal overweight and obesity using various cutoff points to define obesity, and have linked them to only one outcome of interest. This review extensively examines any MNCH outcomes that have been reported with all weight categories, grouping the data from individual studies into underweight or overweight and comparing these to women with normal BMI as defined above (please see table for data included here).

**Figure 2:**
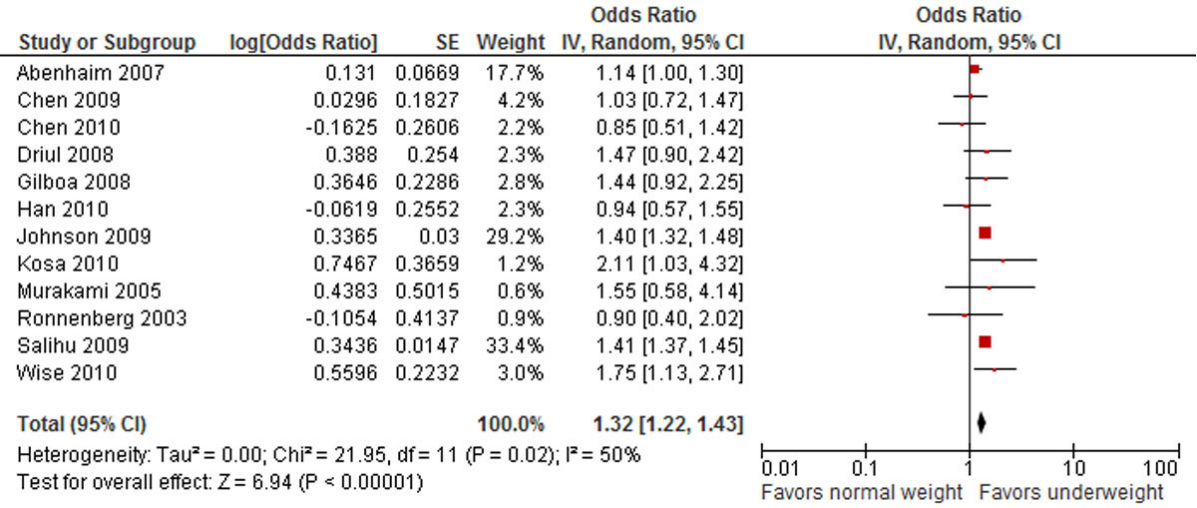
**Pre pregnancy underweight and risk for preterm birth: evidence from observational studies** Citations to the included studies: Abenhaim 2007 [55], Chen 2009 [56], Driul 2008 [57], Johnson 2009 [58], Kosa 2010 [59], Murakami 2005 [60], Ronnenberg 2003 [61], Salihu 2009 [62], Wise 2010[63], Chen 2010 [64], Gilboa 2008 [65], Han 2010 [66].

The review identified 34 studies that discussed maternal underweight [11,13,15,30,31,54-81]. This review found that pre-pregnancy underweight significantly increases the risk of preterm birth by 32% (RR 1.32, 95% CI 1.22-1.43) (Figure 2). Pre-pregnancy underweight was also found to significantly increase the risk of small-for-gestational age babies (RR 1.64, 95% CI 1.22-2.21)., Although previous work has found a significant effect of pre-pregnancy underweight on the risk of having low birth weight babies (RR 1.64 [82] and OR 1.82 [83], this review found a non-significant risk (RR 1.37, 95% CI 0.46-4.13) perhaps because of the low number of studies included since these were the only ones to assess maternal weight status before pregnancy. No effect was found for pre-pregnancy underweight on hypertensive disorders of pregnancy, GDM, large-for-gestational age or macrosomia, or any congenital birth defects.

**Figure 3:**
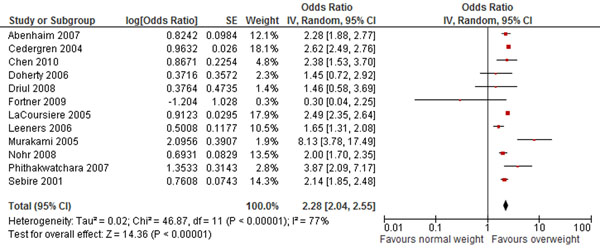
**Pre pregnancy overweight and risk for preeclampsia: evidence from observational studies** Citations to the included studies: Abenhaim 2007 [55], Driul 2008 [57], Fortner 2009 [75], Murakami 2005 [60], Nohr 2008 [67], Cedergren 2004 [29], Chen 2010 [64], Doherty 2006 [11], Leeners 2006 [13], LaCoursiere 2005 [74], Phithakwatchara 2007 [85], Sebire 2001[1]

The review identified 41 studies that reported outcomes for overweight women [1,7,11,13,15,29,54-60,63-67,71-81,84-89]. The results presented in this review for the increased risk of hypertensive disorders of pregnancy with maternal overweight are not truly representative of the association since various studies categorized this outcome differently. The risk of preeclampsia (OR 2.28; 95% CI: 2.04-2.55) (Figure 3) and GDM (OR 1.91; 95% CI: 1.58-2.32), however, approximately doubles with pre-pregnancy overweight, and the effect was even greater for pre-pregnancy obesity. The data for impact of pre-pregnancy obesity is not presented since it is similar to the results for maternal overweight. The Pooled analysis of seven observational studies showed a clear increase of 42% in caesarean delivery among overweight women (OR 1.42, 95% CI 1.21-1.66). This review found inconclusive evidence for the effect of pre-pregnancy overweight on preterm births, instrumental delivery, or fetal distress. Perinatal outcomes that were significantly associated with maternal overweight before conception include macrosomia, large-for-gestational age babies (OR 1.63, 95% CI 1.51-1.76), and birth defects-notably neural tube defects and congenital heart defects (OR 1.15, 95% CI 1.07-1.24).

## Nutritional interventions

### Diet, exercise and weight loss

Consumption of calorie-dense but nutritiously poor foods and physical inactivity are concerning for the health of all people, but disproportionately affect mothers and their young children. Fortunately, weight is a modifiable risk factor and evidence supports weight change as an intervention to improve MNCH outcomes [50,51,56,73,90]. Although this review demonstrated that maternal underweight increases the chances of preterm birth (25%), and small-for-gestational age babies (64%), the review found a scarcity of evidence for interventions to improve the macro-nutritional status of women before pregnancy.

As shown in the previous section, maternal overweight and obesity is a major risk factor for poor maternal and child outcomes. There is some evidence to support exercise as an intervention to decrease the risk of GDM, preeclampsia, and maternal weight gain, improve birth weight, and increase the chance of a normal delivery [91]. This review expands upon previous work [92,93] and examines whether diet and/or exercise are effective in reducing weight in women, and if this impacts MNCH outcomes.

The review 23 identified studies [94-116]. The trials found all used a control group; however they were carried out in women of different ages, and included different interventions. Women in the intervention group lost an average of up to 3.5kg. Interventions that combined calorie restriction and physical activity, involved a support system and monitoring, and were sustained over longer periods effected more weight change. A case control study [96] showed that women with perceived strenuous physical activity before pregnancy had a 78% reduced risk of preeclampsia.

### Folic acid supplementation

Folic acid is a B-vitamin whose bioavailability from dietary sources lags behind that achieved through supplementation, and whose deficiency is associated with congenital abnormalities, especially neural tube defects [117]. Multiple case-control, cohort and quasi-randomised controlled trials have been carried out that provide a strong evidence base to support the effectiveness of folic acid supplementation in preventing birth defects and their consequent morbidity and mortality. Folic acid supplementation has thus become a primary periconceptional intervention.

**Figure 4:**
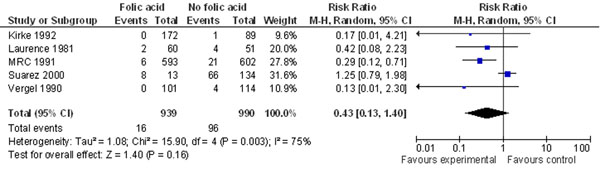
**Folic acid supplementation and risk of recurrent neural tube defects: evidence from observational and experimental studies** Citations to the included studies: Kirke 1992 [119], Laurence 1981 [120], MRC 1991 [118], Suarez 2000 [121], Vergel 1990 [122]

The analyses showed that folic acid has a strong protective effect on preventing recurrent NTDs (RR 0.31, 95% CI 0.14-0.66) when it was restricted to randomized double-blind placebo-controlled studies, however this effect was no longer significant when two observational studies were included- (RR 0.43, 95% CI 0.13-1.40) (Figure 4). The review also found a significant effect of multivitamin supplementation in preventing NTDs, both occurrent and recurrent, although the only included study that did not show a beneficial effect actually separated folic acid from multivitamins, while the rest included folic-acid containing supplements. Hence, it is doubtful that multivitamins without folic acid have a protective effect against NTDs, which is confirmed by the MRC study (RR for multivitamins versus placebo 0.61, 95% CI 0.26-1.45) [118].

Previous reviews have not shown a benefit of folic acid/multivitamin supplementation on orofacial clefts, and although this review added three case-control studies and two prospective cohorts, the effect sizes adhered to unity. However, reviews that include all studies on folic acid/multivitamin supplementation simultaneously do show a modest protective effect [123-126], especially for cleft lip.

**Figure 5:**
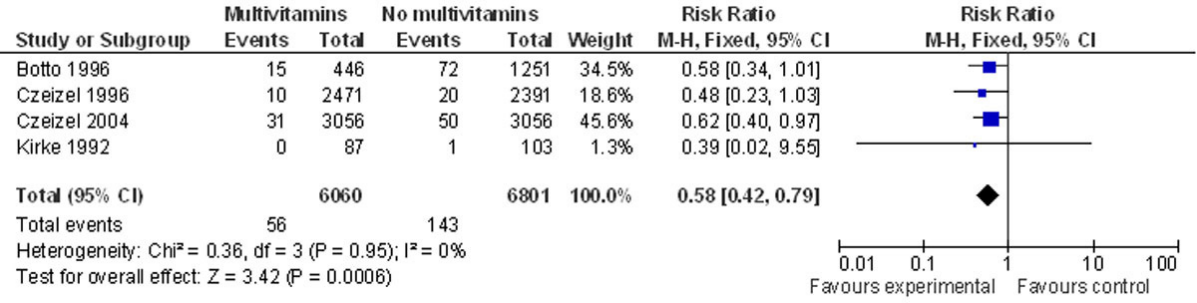
**Folic acid supplementation and risk of congenital heart defects: evidence from observational and experimental studies** Citations to the included studies: Botto 1996 [146], Czeizel 1996 [134], Czeizel 2004 [135], Kirke 1992 [119]

This review identified [118-120,122,127-198] 73 studies on the effect of folic acid supplementation and congenital heart defects. The result is mixed at best- pooling of two randomized trials, one cohort and one case-control trial showed a risk reduction of 42% in this analysis (Figure 5).

No review has shown a consistent effect of folic acid/multivitamin supplementation on maternal and pregnancy outcomes- including ectopic pregnancy, miscarriage, stillbirths, preterm births, low birth weight, and other birth defects. Further, the apprehension that widespread folic acid supplementation or fortification would lead to increased rates of multiple gestation was not shown to be significant in this review (RR 0.99, 95% CI 0.94-1.05) or previous work (OR 1.02 with supplementation and maximum annual increase in twinning rates of 4.6% with fortification) [199].

The results from three randomized double-blind placebo-controlled studies yield a 69% reduced risk (RR of 0.31, 95% CI 0.14-0.66) for recurrent NTDs with periconceptional folic acid supplementation. The MRC study probably provides the most accurate estimate for this intervention since it was a multicenter prospective randomized trial [118]. The remaining studies all suffer from low response rates, however, only Suarez et al. 2000 [121] has results inconsistent with the pooled analysis. This could be attributed to recall and selection bias in the study or to primary intake in this population being from dietary sources with lower bioavailability.

### Multivitamins supplementation

There is incontrovertible data to support the routine use of multivitamins by women of reproductive age, to improve their own health as well as their potential mother and child outcomes. Although previous systematic reviews and meta-analyses have analysed the unique role of periconceptional folic acid (versus multivitamins) on MNCH outcomes, they have included only randomised and quasi-randomised trials. In addition, while periconceptional supplementation is in itself an intervention, it would have a greater impact if it were implemented for all women with the potential to become mothers. For limb reduction defects (RR ranges from 0.43-0.59 for all analyses) and congenital urinary tract anomalies (RR ranges from 0.17-0.68 for all analyses), the evidence shows a modest but persistent risk reduction with the use of *multivitamins*, rather than folic acid.

**Figure 6:**
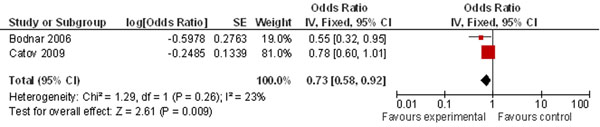
**Multivitamin supplementation and risk of preeclampsia: evidence from observational studies** Citations to the included studies: Bodnar 2006 [155], Catov 2009 [156]

**Figure 7:**
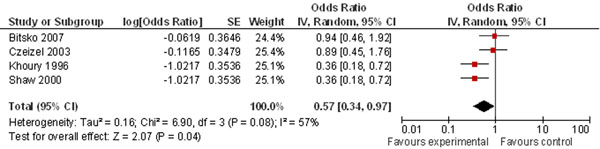
**multivitamin supplementation and risk of multiple congenital anomalies: evidence from observational studies** Citations to the included studies: Bitsko 2007 [149], Czeizel 2003 [150], Khoury 1996 [151], Shaw 2000 [152]

Pooling two cohort studies, the review also found a significant 27% reduction in the risk of preeclampsia (Figure 6) by maternal periconceptional multivitamin supplementation. This review also found a 43% risk reduction of multivitamins for multiple congenital abnormalities (Figure 7).

**Figure 8:**
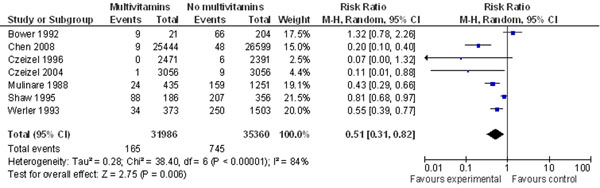
**multivitamin supplementation and risk of concurrent neural tube defects: evidence from observational studies** Citations to the included studies: Bower 1992 [129], Chen 2008[133], Czeizel 1996 [134], Czeizel 2004 [135], Mulinare 1988 [136], Shaw 1995 [200], Werler 1993 [138]

Pooling data from 5 case-control and 2 cohort-controlled trials conducted in relatively high-income countries (HICs), resulted in a significant 49% decrease in risk (RR 0.51, 95% CI 0.31-0.82) (Figure 8) of occurrent neural tube defects due to periconceptional multivitamin supplementation. The case-control study by Bower & Stanley 1992 was inconsistent with the other study results, possibly owing to the small total number of women using periconceptional multivitamins, or to recall bias among case mothers [129]. There was substantial heterogeneity between studies, however repeat analysis using random effects did not change the results.

A recent community intervention in China [201] provided multivitamins to the intervention group from 3 months pre-pregnancy until the end of the first trimester. The intervention resulted in lower incidence of stillbirth (0.70% in intervention group vs. 1.55% in control group; p <0.001), malformation (0.23% in intervention group vs. 0.70% in control group; p <0.001) and low birth weight (0.39% in intervention group vs. 0.84% in control group; p <0.001) compared to the control group, and better growth indicators at birth.

### Iron supplementation

Anemia is a common problem among women of reproductive age, especially in low and middle income countries (LMICs) where low dietary intake of bioavailable iron combined with endemic infectious diseases such as helminthiasis puts women at increased risk in the preconception period. Low preconception hemoglobin and ferritin levels increase the risk of poor fetal growth and low birth weight [202]. The literature shows that iron supplementation during pregnancy can be a protective factor against low birth weight [203], and given alone or with folic acid it is effective in increasing iron stores and preventing anemia during later gestation [204].

The review identified 6 studies [205-210]. Berger et al. [205] tested a weekly combined iron-folic acid intervention in the preconception period among Vietnamese women and similarly found the supplementation significantly improved iron status and reduced anemia when compared to baseline. In the Philippines ferritin levels improved, however hemoglobin lagged behind, possibly due to women being deficient in other micronutrients related to heme formation which were not supplemented during the study [206]. The results of these interventions on a national level were similar to a previous randomized-controlled trial in Bangladesh [207] where iron-folic acid given as a powdered supplement added to food decreased anemia among non-pregnant women; however this benefit did not extend to those women who became pregnant. Recent trials in Vietnam [208,209] combined iron-folic acid supplementation with intermittent deworming and demonstrated significant reduction in anemia, lower rates of helminthic infection, high compliance, and increase in birth weight in intervention districts versus control. In India, the country with the world’s highest proportion of maternal anemia, the same intervention was carried out in adolescent girls, resulting in a substantial drop in anemia prevalence [210].

## Discussion

Maternal overweight and obesity is a growing problem across the world, but women in LMICs and lower socioeconomic strata continue to be at risk of undernourishment [211]. Both pre-pregnancy overweight and underweight are risk factors for poor maternal and child health outcomes, however overweight and obesity results in significantly greater health risks and associated costs. This review identified the association of maternal underweight with preterm birth and small for gestational age babies and the findings are comparable to previous meta-analysis which showed an increased risk of 29% [212] and another study which showed an increased risk of 37% [62]. Similarly the results on association of pre-pregnancy overweight on pre-eclampsia and GDM is consistent with previous reviews that show the risk of preeclampsia typically doubles for each 5 to 7 kg/m^2^ increase in BMI [213]; and the OR of developing GDM is 1.97-2.14 for overweight women, and 3.01-3.56 for obese women [16,214]. The pooled analysis on risk of caseation section and other outcomes are also consistent with previous reviews [2,6,16,215,216]. Given that weight is a modifiable risk factor, research must now focus on how healthcare interventions and public health campaigns can reduce these risks.

There is a strong need for evidence to demonstrate the effectiveness of interventions to achieve optimal pre-pregnancy weight, especially for those women who are underweight. This review confirms earlier evidence [217] that promoting improvement in diet and exercise through sustained, daily changes, with the help of a support system results in weight loss and higher levels of physical activity. Although preceding work illustrates examples of population-scale interventions, more research is needed to support how small-scale initiatives targeted at women with childbearing potential can be implemented on a wider scale. In HICs countries, obese women are increasingly opting to undergo weight loss surgeries, and a review of laparoscopic adjustable gastric banding (LAGB) shows lower gestational weight gain and better maternal and neonatal outcomes for these women compared to obese women not undergoing LAGB prior to pregnancy; however the outcomes were not improved compared to women of normal weight [218].

The role of nutrition in promoting health is well defined. What women eat determines more than just their own health, it is also vital to healthy pregnancies and newborns, and in fact research now shows that nutritional status in early childhood affects health throughout life. Pregnancy, or planning for pregnancy, provides an impetus for women to change non-healthful behaviors. Many women are still unaware of how much their nutritional status impacts their pregnancy outcomes, and improving women’s nutrition and weight-related behaviors should therefore begin during their earlier reproductive years.

Folic acid supplementation has been proven to reduce the risk of NTDs, both recurrent and occurrent and the results are confirmed by the meta-analyses undertaken by De-Regil et al. [37] However, further research is needed to show whether this benefit extends to prevention of orofacial clefts and congenital cardiovascular abnormalities. Although major health organizations promote the use of folic acid by women of reproductive age through clinical guidelines and recommendations [219], and the prevalence of folic acid use is reportedly high in the prenatal period, most women do not use folic acid in the periconceptional period, even if they are aware of its benefits. A recent systematic review [41] demonstrated that even in high income countries, only half of all women use folic acid before conception, therefore protective levels cannot be achieved before the critical period of neural tube closure. Reasons for low prevalence of use are confirmed by other studies [220-228] and include low maternal education and socioeconomic status; young maternal age; lack of a partner; and unplanned pregnancy. It is necessary therefore to improve awareness and use of folic acid supplements among all women of reproductive age so that even women with unplanned pregnancies are protected.

Multicomponent interventions increase use transiently and do not achieve universal coverage, although those with personal counseling in addition to mass campaigns have been shown to be more effective [229]. Fortification has thus been proposed as a means to prevent approximately half of all NTDs occurring annually and 13% of neonatal mortality attributed to NTDs [40], especially in areas with high prevalence of NTDs [230-232]. However, ongoing efforts must be made to supplement women at risk of a recurrent NTDs and women who are more folate-depleted [233,234]. A novel idea has been to incorporate folate into contraceptive pills, which also helps to bridge the gap between when a woman discovers she is pregnant and neural tube closure, even without periconceptional folate use [235]. In order to provide all women (including those at risk of recurrence) with an adequate dose of folic acid, public health policy in some countries now mandates that staple foods, such as flour, be fortified with folic acid.

The studies on iron demonstrate that large-scale nutritional intervention is feasible in LMICs contexts, and results in better biochemical indices. Given the global magnitude of maternal anemia, however, it is surprising that only one trial further assessed birth weight as a measure of improved maternal and newborn health. Iron fortification of foods such as flour, rice, sugar, juice, and fish or soy sauce in various countries has also been shown to improve iron status among women of reproductive age [236-240], but again the analyses of the fortification trials do not assess pregnancy outcomes in the long-term. As with folic acid, preventive iron supplementation may require greater community mobilization and social marketing for increased effectiveness [205] and to contribute to improved women’s and maternal health in developing regions.

## Conclusion

Maternal malnutrition remains a serious global health issue, particularly in LMICs. The median prevalence of low body mass index among women in the preconception period is 10.9% among 24 countries with a recent Demographic and Health Survey, while 42% of women are anemic when they become pregnant [241]. Underweight and deficiencies of essential nutrients coupled with the increasing burden of obesity have consequences during pregnancy and for newborns. These negative effects are amplified in adolescents or women with closely-spaced pregnancies since they have depleted nutritional reserves, which results in stillbirths, neonatal deaths, low birth weight and preterm births [242]. Overweight and obesity further predispose to maternal hypertensive disorders and gestational diabetes.

Among nutrition-specific interventions periconceptional folic acid supplementation significantly reduces the risk of recurrent NTDs. There is growing interest in multiple micronutrient supplementation in at-risk populations in whom multiple deficiencies often coexist. Data for multiple micronutrient supplementation from a small number of controlled trials shows a persistent lowering of rates of congenital anomalies and preeclampsia. Other nutrition-specific interventions (iron, calcium, balanced protein energy supplementation) have only been studied in pregnant women, or if they have been studied during the preconception period the outcomes are limited to changes in biochemical markers while pregnancy and birth outcomes were not assessed.

Strategies for implementation of nutrition-specific interventions in the preconception period are needed especially to reach women in low- and middle-income countries. At present, food fortification with micronutrients is noted to be the most cost-effective large scale method. However, different approaches are needed to specifically increase uptake among women of reproductive age, noting the critical links between poor maternal nutritional status and its wide-ranging determinants and consequences. Nutritional-sensitive interventions improve population health, education and development and countries investing in such strategies have had greater gains in both nutrition and health outcomes. Integrating nutrition with maternal and child health initiatives and developing community-based platforms that are able to reach populations are especially promising.

## Competing interests

We do not have any financial or non-financial competing interests for this review.

## Peer review

Peer review reports are included in additional file 1.

## Supplementary Material

Additional file 1 Peer review reports. Click here for file

## Declarations

This article has been published as part of *Reproductive Health* Volume 11 Supplement 2, 2014: Preconception interventions. The full contents of the supplement are available online at http://www.reproductive-health-journal.com/supplements/11/S3. Publication charges for this collection were funded by the Partnership for Maternal, Newborn & Child Health (PMNCH).
