# Supplementary material for: Preconception care: nutritional risks and interventions
Source: Reprod Health. 2014 Sep 26;11(Suppl 3):S3. doi: 10.1186/1742-4755-11-S3-S3 (PMC4196560; doi:10.1186/1742-4755-11-S3-S3)
Supplement: Additional file 1 — Peer review reports. [file 1742-4755-11-S3-S3-S1.pdf]

Referee's comments to the authors– this sheet WILL be seen by the author(s) and published with the article

Title	**Preconception care: nutritional status and nutritional interventions**
Author(s)	Sohri V Dean, Zohra S Lassi, Ayesha M Imam, Zulfiqar A Bhutta
Referee's name	Jai Das

When assessing the work, please consider the following points, where applicable:

1. Is the question posed by the authors new and well defined?
2. Are the methods appropriate and well described, and are sufficient details provided to replicate the work?
3. Are the data sound and well controlled?
4. Does the manuscript adhere to the relevant standards for reporting and data deposition?
5. Are the discussion and conclusions well balanced and adequately supported by the data?
6. Do the title and abstract accurately convey what has been found?
7. Is the writing acceptable?

Please make your report as constructive and detailed as possible in your comments so that authors have the opportunity to overcome any serious deficiencies that you find and please also divide your comments into the following categories:

- Major Compulsory Revisions (which the author must respond to before a decision on publication can be reached)
- Minor Essential Revisions (such as missing labels on figures, or the wrong use of a term, which the author can be trusted to correct)
- Discretionary Revisions (which are recommendations for improvement but which the author can choose to ignore)

Where possible please supply references to substantiate your comments.

When referring to the manuscript please provide specific page and paragraph citations where appropriate.

(continue on the next sheet)

General comments:

The authors have identified an important area and done a good job in reviewing the area but have failed to justify the effort while presenting the findings, the papers needs a lot of work in terms of structuring and organizing the work that they have carried out.

Major compulsory revisions:

The title is vague; it should reflect the contents which are the nutritional risks and interventions for women in the preconception period.

The background NEVER emphasizes the rationale of nutrition interventions in the preconception period and a need to do this review. Background should reflect what the authors intend to do and linked to the current knowledge and a need to intervene in this period.

The objective is not mentioned in the background, methods or even the title doesn't suggest it, I understand that it is part of a series but still the paper in itself should be stand-alone and reflect the intentions of doing this review.

Methods: not adequately described, is it a systematic review or a review of reviews. 'Standard methodology' should be referenced.

Results:

The authors suddenly jump to the results without giving an idea to the readers what they should expect and there is a lot of material which should be ideally placed in the background rather than the results and if they cannot all be presented in the background and results should be structured so that there are separate sections for the existing evidence and the evidence from the review.

The interventions results and risk factors are all described together, I would suggest reorganize the results in a way so that risk factors and intervention impacts are described separately, this would do justice with the work that has been carried out.

A brief description of the characteristics of the included studies is warranted like age group, settings, developing or developed countries, exact intervention. The draft says varying intervention and then go on to give an estimate without mentioning the variances in intervention

There should be a table summarizing the results of the interventions/risks reviewed

Minor essential revisions:

Abstract

MNCH not defined in the abstract

'losely-spaced pregnancies' should mention the time period

Introduction

Page 2 1st paragraph- 'Nutritional status is a prime component of health and wellness before and during pregnancy. This includes weight, stature, dietary patterns and nutritional supplements' - Component is not an appropriate word and dietary patterns is not reflective of nutrition status

The first reference is old and new data is available, so authors should update the estimates

In the last sentence of the first paragraph the authors seem to portray that changing genetics and age at first pregnancy are equally difficult to change, which is obviously not correct, this statement need rephrasing

The background should end with the objective and goal of the paper, which is not mentioned

The phrase 'double burden is used too many times in the draft'

Methods

not at all adequately described and mentioning' Standard criteria were applied to determine the quality of the evidence, and standard methods for meta-analyses of quantitative studies were used' authors should provide reference of the standard methods and if the methods are described in detail in another paper then it should be mentioned and the paper should be referenced.

Inclusion/exclusion should be clearly mentioned.

Continued

Results
WHO classification of weight needs a reference
The authors should further describe why only two studies were included for impact on SGA and how many studies did Salihu 2009 include and what the rationale behind including less studies.
Again for LBW the authors state that conflicting results were due to less number of studies being included but failed to describe why there were less studies included than other review- was it because of the study quality or stringent inclusion criteria, this should be stated so that readers can have an idea what the correct relation is.
'As expected', not a word to use for the results
'obesity although this data was not presented as meta-views.' Then how was it presented
'Maternal overweight and obesity is a growing problem across the world, but women in developing countries and lower socioeconomic strata continue to be at risk of undernourishment [86]. Both prepregnancy overweight and underweight are risk factors for poor maternal and child health outcomes, however overweight and obesity results in significantly greater health risks and associated costs. Given that weight is a modifiable risk factor, research must now focus on how healthcare interventions and public health campaigns can reduce these risks' - at the end of the results the authors describe that obesity is a growing problem, which has been or should be done in the previous sections and at the end of the results should be concluding statement regarding the findings of the review itself.
MNCH- full form
'23 identified studies; should be 'identified 23 studies',
Figure of search strategy just shows the total number of studies included, it would be better if we could subgroup it according to risks/intervention
Discretionary revisions:
:

Referee's comments to the authors– this sheet WILL be seen by the author(s) and published with the article

Title	**Preconception care: nutritional status and nutritional interventions**
Author(s)	Sohri V Dean, Zohra S Lassi, Ayesha M Imam, Zulfiqar A Bhutta
Referee's name	Usha Ramakrishnan

When assessing the work, please consider the following points, where applicable:

1. Is the question posed by the authors new and well defined?
2. Are the methods appropriate and well described, and are sufficient details provided to replicate the work?
3. Are the data sound and well controlled?
4. Does the manuscript adhere to the relevant standards for reporting and data deposition?
5. Are the discussion and conclusions well balanced and adequately supported by the data?
6. Do the title and abstract accurately convey what has been found?
7. Is the writing acceptable?

Please make your report as constructive and detailed as possible in your comments so that authors have the opportunity to overcome any serious deficiencies that you find and please also divide your comments into the following categories:

- Major Compulsory Revisions (which the author must respond to before a decision on publication can be reached)
- Minor Essential Revisions (such as missing labels on figures, or the wrong use of a term, which the author can be trusted to correct)
- Discretionary Revisions (which are recommendations for improvement but which the author can choose to ignore)

Where possible please supply references to substantiate your comments.

When referring to the manuscript please provide specific page and paragraph citations where appropriate.

General comments:
This paper addresses an important topic on the importance of preconception care with respect to nutrition. However, it lacks details with respect to the methods that make it difficult to evaluate the contribution in addition to what has been published to date. The presentation of the results is also confusing and need clarification. The results include many statements that reflect the opinions of the authors and do not belong to this section. There are also several errors indicating that this has not been read carefully and statements that are not supported by relevant citations.

Major compulsory revisions:

Background: The authors should cite recent reviews on this topic and clarify the scope of the review.

Methods: The authors do not provide details of the search terms used for the systematic review and inclusion and exclusion criteria that were used to identify eligible studies. Specifically, they show that several studies were excluded at various stages but it is unclear why and how they arrived with the final samaple of 146 studies. The rationale for conducting meta-analyses has also not been provided especially since for certain areas these have already been conducted**.**

(continue on the next sheet)

Continued:

Results: The presentation of the findings is very confusing and difficult to follow. There are several sections where the authors have included text that would typically belong with the discussion. A few examples are described below to illustrate this point but all sections need to be revised appropriately.

Maternal pre-pregnancy weight: This section begins with a summary of some of the findings of the risks of being overweight and/or obese, switches to underweight and again reverts of overweight in the following page. The authors provide several relative risks but it is unclear which studies contributed to the calculation of these estimates making it difficult to evaluate and interpret their findings. For example, it would be helpful to know if they have included more studies compared to the previous reviews etc. and/or excluded some studies. The last paragraph does not belong to the results. A table that summarises the key findings by exposure and outcome of interest with the relevant references is recommended.

Diet, exercise and weight loss: The first and last 2 paragraphs do not belong to the results section. The authors also state thaty they find only two studies that evaluated the benefit of physical activity before pregnancy but cite only one study.

Folic acid: Once again, the first few paragraphs do not belong to the results. It is also unclear why the authors combined results from intervention and observational studies when so many intervention trials have been done.

Multivitamins: The authors should provide details on what vitamins were provided for the different studies and what the comparison groups received. They also should clarify if the evidence is based on controlled trials versus observational studies. For example, several studies included in Figures 3 and 7 are intervention trials whereas others are based on observational studies. The authors comment on one recent large study in China that reported birth outcomes such as preterm delivery, stillbirth and low birth weight abut do not provide details on the magnitude of the effects and nature of the intervention.

Conclusions: The authors do not cite relevant publications to support many of their statements in the first two paragraphs. For example, they mention the increased risk of poor outcomes among women with closely spaced pregnancies and/or adolescent women but fail to cite the recently published systematic reviews. They should also clearly state the gaps in the literature and relevant recommendations for future research.

Minor Essential Revisions

There are several typographic errors that should be corrected.

Results-

Folic Acid – the first sentence in the 8th paragraph is incorrect and should state “ This review identified 73 studies [139-214]”.

Iron – The first sentence in the second paragraph of this section should read “ The review……..”.
The last sentence in this paragraph is also missing a period at the end.

Referee’s comments to the authors– this sheet WILL be seen by the author(s) and published with the article

Title	**Preconception care: nutritional status and nutritional interventions**
Author(s)	Sohri V Dean, Zohra S Lassi, Ayesha M Imam, Zulfiqar A Bhutta
Referee’s name	Jai Das

When assessing the work, please consider the following points, where applicable:

1. Is the question posed by the authors new and well defined?
2. Are the methods appropriate and well described, and are sufficient details provided to replicate the work?
3. Are the data sound and well controlled?
4. Does the manuscript adhere to the relevant standards for reporting and data deposition?
5. Are the discussion and conclusions well balanced and adequately supported by the data?
6. Do the title and abstract accurately convey what has been found?
7. Is the writing acceptable?

Please make your report as constructive and detailed as possible in your comments so that authors have the opportunity to overcome any serious deficiencies that you find and please also divide your comments into the following categories:

- Major Compulsory Revisions (which the author must respond to before a decision on publication can be reached)
- Minor Essential Revisions (such as missing labels on figures, or the wrong use of a term, which the author can be trusted to correct)
- Discretionary Revisions (which are recommendations for improvement but which the author can choose to ignore)

Where possible please supply references to substantiate your comments.

When referring to the manuscript please provide specific page and paragraph citations where appropriate.

(continue on the next sheet)

General comments:
The authors have identified an important area and done a good job in reviewing the area but have failed to justify the effort while presenting the findings, the papers needs a lot of work in terms of structuring and organizing the work that they have carried out.

Major compulsory revisions:
The title is vague; it should reflect the contents which are the nutritional risks and interventions for women in the preconception period.
Title amended “Preconception Care: nutritional risks and interventions”

The background NEVER emphasizes the rationale of nutrition interventions in the preconception period and a need to do this review. Background should reflect what the authors intend to do and linked to the current knowledge and a need to intervene in this period.
We have improved the background.

The objective is not mentioned in the background, methods or even the title doesn’t suggest it, I understand that it is part of a series but still the paper in itself should be stand-alone and reflect the intentions of doing this review.
Added “This paper presents the findings of a systematic review that was undertaken to consolidate the evidence for nutritional risks before pregnancy, and ascertain the effectiveness of providing interventions during the preconception period on maternal, newborn and child health (MNCH) outcomes.”

Methods: not adequately described, is it a systematic review or a review of reviews. ‘Standard methodology’ should be referenced.
Methods are now more explicit and reference to detailed methods in paper 1 added

Results:
The authors suddenly jump to the results without giving an idea to the readers what they should expect and there is a lot of material which should be ideally placed in the background rather than the results and if they cannot all be presented in the background and results should be structured so that there are separate sections for the existing evidence and the evidence from the review.
Sections not for results moved to background or discussion as appropriate.

The interventions results and risk factors are all described together, I would suggest reorganize the results in a way so that risk factors and intervention impacts are described separately, this would do justice with the work that has been carried out.
We have now categorized them as Nutritional risk and Nutritional interventions

A brief description of the characteristics of the included studies is warranted like age group, settings, developing or developed countries, exact intervention. The draft says varying intervention and then go on to give an estimate without mentioning the variances in intervention
We can hyperlink the 2 tables for interventions for weight control and folic acid supplementation. These give intervention characteristics and outcoms
https://globalmotherchildresearch.tghn.org/site_media/media/articles/Preconception_Report.pdf

There should be a table summarizing the results of the interventions/risks reviewed
We do not feel the importance of having this table. As these have been described in results and in forest plots. Also have been discussed in the discussion section.

Minor essential revisions:
Abstract
MNCH not defined in the abstract Added
‘closely-spaced pregnancies’ should mention the time period Added “(inter-pregnancy interval less than six months)

Introduction
Page 2 1st paragraph- ‘Nutritional status is a prime component of health and wellness before and during pregnancy. This includes weight, stature, dietary patterns and nutritional supplements’ - Component is not an appropriate word and dietary patterns is not reflective of nutrition status
Amended to “Nutritional status is an important aspect of health and wellness before and during pregnancy.”

Continued

In the last sentence of the first paragraph the authors seem to portray that changing genetics and age at first pregnancy are equally difficult to change, which is obviously not correct, this statement need rephrasing

Rephrased to "Weight and micronutrient status during pregnancy is influenced by a number of factors such as food insecurity and birth spacing that require broad interventions, hence the aim should to achieve and sustain optimal nutritional intake and weight before pregnancy."

The background should end with the objective and goal of the paper, which is not mentioned

Added

The phrase 'double burden is used too many times in the draft'

Removed

Methodsnot at all adequately described and mentioning' Standard criteria were applied to determine the quality of the evidence, and standard methods for meta-analyses of quantitative studies were used' authors should provide reference of the standard methods and if the methods are described in detail in another paper then it should be mentioned and the paper should be referenced. Inclusion/exclusion should be clearly mentioned.

We have now describved the methods part in detail.

:

Results

WHO classification of weight needs a reference Provided.

The authors should further describe why only two studies were included for impact on SGA and how many studies did Salihu 2009 include and what the rationale behind including less studies.

We have modified the section and since this was suggested to be removed from results to discussion, this section is moved to discussion. On modification of text this sentence has been removed.

Again for LBW the authors state that conflicting results were due to less number of studies being included but failed to describe why there were less studies included than other review- was it because of the study quality or stringent inclusion criteria, this should be stated so that readers can have an idea what the correct relation is.

Explained that others not included because did not adhere to the preconception period timeframe

‘As expected’, not a word to use for the results
Changed

‘obesity although this data was not presented as meta-views.’ Then how was it presented
Explained that since these results could be extrapolated from the meta-views for overweight, it was chosen to not present the same data twice (albeit with increased magnitude of impact for obesity in general)

‘Maternal overweight and obesity is a growing problem across the world, but women in developing countries and lower socioeconomic strata continue to be at risk of undernourishment [86]. Both prepregnancy overweight and underweight are risk factors for poor maternal and child health outcomes, however overweight and obesity results in significantly greater health risks and associated costs. Given that weight is a modifiable risk factor, research must now focus on how healthcare interventions and public health campaigns can reduce these risks’ - at the end of the results the authors describe that obesity is a growing problem, which has been or should be done in the previous sections and at the end of the results should be concluding statement regarding the findings of the review itself.
Removed

MNCH- full form full form has been used in the first place in the text before the abbreviation

‘23 identified studies; should be ‘identified 23 studies’, Corrected

Figure of search strategy just shows the total number of studies included, it would be better if we could subgroup it according to risks/intervention
We have now subdivided results as nutritional risks and interventions. We hope this is now clear.

Discretionary revisions:

Referee's comments to the authors– this sheet WILL be seen by the author(s) and published with the article

Title	**Preconception care: nutritional status and nutritional interventions**
Author(s)	Sohri V Dean, Zohra S Lassi, Ayesha M Imam, Zulfiqar A Bhutta
Referee's name	Usha Ramakrishnan

When assessing the work, please consider the following points, where applicable:

1. Is the question posed by the authors new and well defined?
2. Are the methods appropriate and well described, and are sufficient details provided to replicate the work?
3. Are the data sound and well controlled?
4. Does the manuscript adhere to the relevant standards for reporting and data deposition?
5. Are the discussion and conclusions well balanced and adequately supported by the data?
6. Do the title and abstract accurately convey what has been found?
7. Is the writing acceptable?

Please make your report as constructive and detailed as possible in your comments so that authors have the opportunity to overcome any serious deficiencies that you find and please also divide your comments into the following categories:

- Major Compulsory Revisions (which the author must respond to before a decision on publication can be reached)
- Minor Essential Revisions (such as missing labels on figures, or the wrong use of a term, which the author can be trusted to correct)
- Discretionary Revisions (which are recommendations for improvement but which the author can choose to ignore)

Where possible please supply references to substantiate your comments.

When referring to the manuscript please provide specific page and paragraph citations where appropriate.

General comments:
This paper addresses an important topic on the importance of preconception care with respect to nutrition. However, it lacks details with respect to the methods that make it difficult to evaluate the contribution in addition to what has been published to date. The presentation of the results is also confusing and need clarification. The results include many statements that reflect the opinions of the authors and do not belong to this section. There are also several errors indicating that this has not been read carefully and statements that are not supported by relevant citations.
We have now revisited all the sections and have improved them accordingly.

Major compulsory revisions:

Background: The authors should cite recent reviews on this topic and clarify the scope of the review.
We have made changes in the background and cited recent reviews.

Methods: The authors do not provide details of the search terms used for the systematic review and inclusion and exclusion criteria that were used to identify eligible studies. Specifically, they show that several studies were excluded at various stages but it is unclear why and how they arrived with the final samaple of 146 studies. The rationale for conducting meta-analyses has also not been provided especially since for certain areas these have already been conducted.
Added these components and methods in detail. However, the search terms are provided in Paper 1 and we have given the reference to that paper in the methods part.

Details of the electronic databases should be provided
Same as above.

Continued:

Results: The presentation of the findings is very confusing and difficult to follow. There are several sections where the authors have included text that would typically belong with the discussion. A few examples are described below to illustrate this point but all sections need to be revised appropriately.
Removed those texts from results and added in discussion.

Maternal pre-pregnancy weight: This section begins with a summary of some of the findings of the risks of being overweight and/or obese, switches to underweight and again reverts of overweight in the following page. The authors provide several relative risks but it is unclear which studies contributed to the calculation of these estimates making it difficult to evaluate and interpret their findings. For example, it would be helpful to know if they have included more studies compared to the previous reviews etc. and/or excluded some studies. The last paragraph does not belong to the results. A table that summarises the key findings by exposure and outcome of interest with the relevant references is recommended.
We have now hyperlinked the table to the review at the appropriate place, and mentioned that this review was significantly more extensive than previous reviews since it examined all weight categories as defined by individual studies, grouped them into 3 categories of maternal preconception weight (underweight, normal BMI, overweight including obese with BMI >25) and linked them with all reported MNCH outcomes

Diet, exercise and weight loss: The first and last 2 paragraphs do not belong to the results section. The authors also state that they find only two studies that evaluated the benefit of physical activity before pregnancy but cite only one study.
Corrected and removed from there.

Folic acid: Once again, the first few paragraphs do not belong to the results. It is also unclear why the authors combined results from intervention and observational studies when so many intervention trials have been done.
Paragraphs that fit better in the introduction or discussion have been moved. We aimed to dissect the effect of folic acid separately from other multivitamins, to assess any folate versus no folate us ALL in the preconception (versus periconception or prenatal) period, hence we combined results from intervention and observational studies. These suggest that folic acid alone in the preconception period has positive effects on neural tube defects, however multivitamin supplementation may result in lower rates of orofacial clefts.

Multivitamins: The authors should provide details on what vitamins were provided for the different studies and what the comparison groups received.
We can provide the link to the report where all studies are described in detail.
They also should clarify if the evidence is based on controlled trials versus observational studies. For example, several studies included in Figures 3 and 7 are intervention trials whereas others are based on observational studies. We have mentioned them next to their respective plot. Because this was also suggested for other paper from the series. The authors comment on one recent large study in China that reported birth outcomes such as preterm delivery, stillbirth and low birth weight abut do not provide details on the magnitude of the effects and nature of the intervention. Added

Conclusions: The authors do not cite relevant publications to support many of their statements in the first two paragraphs. For example, they mention the increased risk of poor outcomes among women with closely spaced pregnancies and/or adolescent women but fail to cite the recently published systematic reviews. They should also clearly state the gaps in the literature and relevant recommendations for future research.
Conclusion is now separated from discussion. The discussion section cites the relevant systematic reviews also we have added gaps and recommendations for future studies.

Minor Essential Revisions

There are several typographic errors that should be corrected.

Results-

Folic Acid – the first sentence in the 8th paragraph is incorrect and should state “ This review identified 73
